# Minimalist Footwear in the Treatment and Rehabilitation of Lower Limb Impairments Across the Life Course: A Scoping Review

**DOI:** 10.1002/msc.70122

**Published:** 2025-05-24

**Authors:** Stewart C. Morrison, Ben Langley, Binyu Luo, Carina Price

**Affiliations:** ^1^ Faculty of Life Sciences and Medicine King's College London London UK; ^2^ Department of Sport and Physical Activity Edge Hill University Ormskirk UK; ^3^ School of Health and Society University of Salford Salford UK

**Keywords:** barefoot, foot, intervention, musculoskeletal, shoe

## Abstract

**Background:**

Minimalist footwear has emerged as an alternative to traditional footwear styles and advocated for the management of several foot and lower limb pathologies.

**Objective:**

The objective of this scoping review was to map the clinical potential of minimalist footwear (concept) in the treatment and/or rehabilitation of lower limb impairments (context) across the life course (population).

**Data Sources:**

Systematic searches were undertaken across MEDLINE, EMBASE, and CINAHL from 2000 to 2024.

**Study Selection or Eligibility Criteria:**

Studies evaluating minimalist footwear as an intervention or adjunct to an intervention in clinical populations, or where a clinical need has been defined, across all age groups, were included. Eligible studies were primary research published in English from the year 2000 onwards.

**Data Synthesis:**

A narrative analysis was undertaken and our findings were reported in accordance with the PRISMA‐ScR guidelines.

**Results:**

Sixteen studies were identified in clinical populations ranging from adolescents with patello‐femoral pain (14.3; SD: 1.7 years) to older adults with balance ability (73.4; SD: 3.9 years); studies focussing on knee pathology were the most common (*n* = 9). The influence of minimalist footwear on outcome measures varied across the studies and reported mechanisms of action included somatosensory, biomechanical and neuromuscular factors.

**Conclusion and Implications:**

Our review has mapped the clinical populations where minimalist footwear has been tested and most focus on knee pathology, specifically knee osteoarthritis. Our review has identified the biomechanical, functional, and clinical variables reported in studies and future work testing the clinical benefits of minimalist footwear interventions is recommended.

**Abbreviations**
DOI: digital object identifier
EKAM: external knee adductor moment
EVA: ethylene‐vinyl acetate
IMU: inertial measurement units
KAAI: knee adduction angular impulse
PF: patello‐femoral
PFJ: patello‐femoral joint
PRISMA: preferred reporting items for systematic reviews and meta‐analysis
WOMAC: Western Ontario and McMaster Universities Index

## 1 Introduction

Footwear interventions are an important consideration for people experiencing foot and lower limb problems (Brenton‐Rule et al. 2019; Hatton and Rome 2019; McRitchie et al. 2018; Menz et al. 2017) and many studies documenting the impact of footwear across the life course exist. Whilst not an exhaustive overview, studies have documented the impact of footwear on foot development in children (Morrison et al. 2018; Price et al. 2021; Wang et al. 2023), discussed the role of footwear on falls risk in older adults (Hatton and Rome 2019; Menz et al. 2024; Menz et al. 2017), described how footwear can support function in adults with long‐term conditions (Frecklington et al. 2019; Novo‐Trillo et al. 2021), and influence performance (Fuller et al. 2015; Nesterovica et al. 2021). Studies report a range of parameters including biomechanical (e.g. movement, loading and strength parameters), functional (e.g. balance and mobility measures) and clinical (e.g. pain measures) but there is little consistency across the evidence. There is a debate about the role of footwear in the treatment and/or modification of factors that contribute to injury; findings from a recent Cochrane review established that footwear choice did not reduce lower limb injury in runners (Relph et al. 2022). The extent to which this argument extends beyond the running context is unclear, but nonetheless, it is recognised that footwear choice is not without risk (Azhar et al. 2023). In some instances, barefoot (or minimalist footwear) conditions may be preferable (Hollander et al. 2022; Ren et al. 2022), underpinning the debate about the role of footwear on foot morphology and function (D’Août et al. 2009) and calling for functionally individualised footwear (Mai et al. 2023).

Over the past few decades, minimalist footwear has emerged as an alternative to traditional footwear styles. This footwear attempts to promote the natural movement of the foot (Coetzee et al. 2018; Esculier et al. 2015) and minimalist shoes are characterised by high flexibility, low heel‐to‐toe drop, lightweight construction, minimal stack height, and lack of motion control and stability features (Esculier et al. 2015). Studies have demonstrated that minimalist footwear influences biomechanical parameters such as lower limb joint kinematics (Petersen et al. 2020), and, in some individuals, increases (intrinsic) foot muscle strength (Allen et al. 2023; Curtis et al. 2021), leading to suggestions this type of footwear may be an effective intervention or adjunct to intervention in clinical populations (Cudejko, Gardiner, Akpan, and D’Août 2020b; Davis et al. 2021). To date, studies have reported that minimalist shoes can improve stability in people with a history of falls (Allen et al. 2023; Cudejko, Gardiner, Akpan, and D’Août 2020b), relieve pain in older women with knee osteoarthritis (Trombini‐Souza et al. 2015) and improve balance in children (Quinlan et al. 2022). Xu (Xu et al. 2023) proposed that minimalist footwear could be a beneficial adjunct to exercise programmes in clinical populations.

## 2 Review Question

Based upon the Population‐Concept‐Context (PCC) framework, this review will address the following research question: What is the clinical/therapeutic potential of minimalist footwear (concept) in the treatment and/or rehabilitation of lower limb impairments (context) across the life course (population)?

The objectives of the review are to:

1. Synthesise the biomechanical (e.g. movement, loading and strength parameters), functional (e.g. balance and mobility measures) and clinical (e.g. outcome measures) variables reported in studies evaluating the therapeutic potential of minimalist footwear.
2. Map the clinical populations where minimalist footwear has been tested as an intervention or adjunct to an intervention.
3. Outline the reported mechanisms of action of minimalist footwear.

## 3 Method

This scoping review was conducted in accordance with the JBI methodology for scoping reviews (Aromataris et al. 2024) and reported in accordance with the Preferred Reporting Items for Systematic Reviews and Meta‐Analysis (PRISMA) extension for scoping reviews (Tricco et al. 2018). The protocol was registered on the Open Science Framework (DOI: 10.17605/OSF.IO/XPY5E).

The inclusion criteria were defined using the PCC framework:

### 3.1 Participants/Population

This review considered studies across the life course which have evaluated minimalist footwear as an intervention, or adjunct to an intervention, in clinical populations, or where a clinical need has been defined.

### 3.2 Concept

The concept explored was minimalist footwear. Due to variance in the literature, we did not include a fixed definition of minimalist footwear but aligned inclusion with the principles reported by Esculier et al. (Esculier et al. 2015). The key characteristics of minimalist shoes include high flexibility, low heel‐to‐toe drop, lightweight construction, minimal stack height, and the absence of motion control and stability devices (Esculier et al. 2015). The requirement for the concept being met was defined by the reviewers at full‐text screening, identifying footwear meeting the above definition or self‐report from the authors.

### 3.3 Context

Research studies which evaluated the therapeutic potential of minimalist footwear for the treatment and/or rehabilitation of lower limb impairment(s) were considered. The term therapeutic potential was used to capture any study which involved participants where minimalist footwear was used as an intervention, or within an intervention package, to ameliorate the impact of impairment(s) on individuals' function, performance and/or participation. These studies could be proof‐of‐concept laboratory‐based studies (with defined clinical populations) or conducted within the clinical setting (such as randomised control trials). All geographical locations were included.

## 4 Types of Sources

A systematic search was undertaken and included peer‐reviewed, primary research studies of quantitative design, including randomised and non‐randomised clinical trials, analytical and non‐analytical observational design (e.g. cohort, case‐control, cross‐sectional and case study design). We excluded qualitative research, reviews (e.g. narrative or systematic), and conference abstracts as we did not anticipate that they would contain sufficient information to answer the research questions.

## 5 Search Strategy

As defined in the JBI guidance (Aromataris et al. 2024), a three‐step search strategy was adopted. An initial search of MEDLINE and CINAHL was undertaken to identify articles on the topic. The text words contained in the titles and abstracts of these articles, and the index terms, were used to develop a full search strategy, which was developed and agreed with support from an academic librarian. An example search strategy is reported (see Supporting Information S1: appendix 1). The databases searched were MEDLINE, EMBASE, and CINAHL, and the search strategy was adapted for each database. Sources of ongoing and recently completed studies/grey literature were searched on ClinicalTrials.gov (the US National Institute of Health Clinical Trials search portal) and Web of Science.

The reference list of all included sources of evidence was screened for additional studies. If required, the authors were contacted for information about their manuscript(s). We included studies written in English and to ensure a contemporary perspective of the literature, we included studies published from the year 2000 onwards.

## 6 Study/Source of Evidence Selection

**FIGURE 1:**
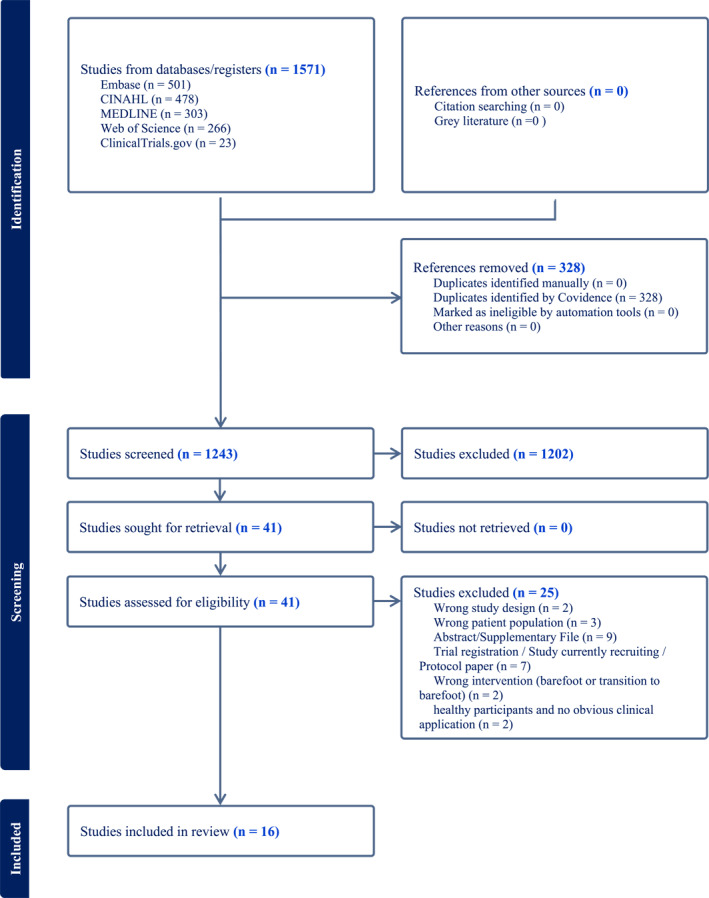
PRISMA flow‐chart.

Citations were collated into Mendeley, and all duplicates removed. The titles and abstracts were independently screened by two reviewers [Binya Luo, Stewart C. Morrison] and the full text of selected citations independently assessed against the inclusion criteria. This was undertaken by the same two reviewers. Exclusion of full‐text sources was recorded and disagreements between the reviewers were discussed. Where there was disagreement, a third reviewer [Ben Langley] was consulted. The results of the search and the study inclusion process are reported in a PRISMA flow diagram (Figure 1) (Tricco et al. 2018).

## 7 Data Extraction

The data extraction form was based on recommendations from the JBI (Pollock et al. 2023) and tailored to reflect the research question. The data extracted from studies included participant demographics, concepts, context and key findings reported in the study and was initially piloted by two reviewers [Ben Langley, Carina Price] on two papers representing different types of research articles. Data extraction was undertaken and charted independently. Following this, there was a discussion with the wider research team and additional information was extracted from each of the studies relating to study inclusion/exclusion criteria, reporting of comorbidities, detailed footwear characteristics, recruitment setting and methods and the specific tasks or activities being undertaken by participants. A narrative synthesis was undertaken.

The PRISMA flowchart for the study is presented in Figure 1. The original search returned 1571 sources, of which 1242 remained after the removal of duplicates.

## 8 Results

A summary of the screening and inclusion of studies in the review is presented (see Figure 1). A total of 1571 studies were imported into Covidence, and 328 duplicates were removed. At the title and abstract screening stage, 1202 studies were excluded and 41 were considered at full‐text screening. Twenty‐five studies were excluded and 16 progressed to data extraction.

### 8.1 Description of Studies

A total of 489 participants were included within the 16 studies (see Table 1). Seven studies reported results for a mixed sex sample (i.e. male and female), 7 reported a female‐only sample, and 2 reported male‐only. The smallest sample size was 1 (case study in hallux valgus) and the largest was 70 (randomised control trial in knee pain). The clinical populations ranged from adolescents with patello‐femoral pain (14.3; SD: 1.7 years) through to older adults (73.4; SD: 3.9 years), with studies focussing on the knee being most common (*n* = 9); knee osteoarthritis (*n* = 6), patello‐femoral pain (*n* = 3). Eleven of the studies were observational (4 cohort studies, 5 cross‐sectional studies, 1 case‐control, and 1 case study) and five of the studies were experimental. Studies were conducted in Australia (*n* = 5), Brazil (*n* = 5), the United Kingdom (*n* = 3), China (*n* = 2) and the United States of America (*n* = 1). Settings varied across the studies and ranged from the clinical setting to the movement laboratory setting, with the latter being the most common (*n* = 9).

**TABLE 1:** Summary of included studies.

Study	Aim	Country	Study design	Participants	Footwear characteristics	Functional tasks undertaken	Data collection & outcomes
Azhar et al. (2023)	To compare balance ability and walking stability in older women while wearing more aesthetically appealing supportive and minimalist footwear, and to investigate older womens' perceptions of the two types of footwear	Australia	Cohort	20 women aged 65 + (mean age 73.4 years; SD 3.9 years)	**Minimalist:** Canvas upper laced pump: Kmart. Shore A = 35, 10 mm heel and 5 mm forefoot rubber sole; mass = 191–258 g **Comparator:** Supportive, casual style laced and velcro fastening: Ziera, Able Health. Shore A = 55, 20 mm heel and 10 mm forefoot. Firm heel counter; mass = 313–342 g; Grooved outsole + Textured insole	Bipedal standing (eyes open and closed) ‘Near’ tandem standing (eyes open) walking (treadmill, flat & irregular surfaces)	QuickScreen (falls risk) IMU (postural sway & walking stability) Monitor orthopaedic shoes questionnaire Footwear comfort scale
Bonacci et al. (2020)	To determine the effect of running with an increased cadence and in a minimalist shoe on lower limb variability in runners with PFP	Australia	Cohort	15 recreational runners with patellofemoral pain; 3 male and 12 female (mean age 32.6 years; SD 9.6 years)	**Minimalist:** Vibram Seeya. 0‐mm heel–toe offset. Stack height of 5 mm, mass = 136 g **Comparator:** Running shoe Asics gel‐cumulus 16. 11‐mm heel–toe offset. Stack height of 31 mm; mass = 345 g	Treadmill running	Instrumented treadmill & 3D motion capture: Movement coupling variability (modified vector coding); lower limb angles and moment variability (approximate entrophy)
Bonacci et al. (2018)	To determine the effect of a combination of a minimalist shoe and increased cadence on measures of patellofemoral joint loading during running in individuals with patellofemoral pain	Australia	Cross‐sectional, repeated measures	15 runners with patellofemoral pain; 3 male and 12 female (mean age 32.6 years; SD 9.6 years)	**Minimalist:** Vibram Seeya. 0‐mm heel–toe offset. Stack height of 5 mm, mass = 136 g **Comparator:** Running shoe Asics gel‐cumulus 16. 11‐mm heel–toe offset. Stack height of 31 mm. Mass = 345 g	Running	Instrumented treadmill and 3D motion capture: Peak PFJ stress; peak PFJRF; peak knee extensor moment and peak knee flexion angle
Cudejko et al. (2020a)	(1) Compare the effects of footwear type on stability and mobility in persons with a history of falls, and (2) determine whether the effect of footwear type on stability is altered by the absence	United Kingdom	Cross‐sectional, repeated measures	30 older adults with a history of falls (mean age: 68.6 years; SD 4.4 years); 17 female, 13 male.	**Minimalist:** Conventional minimalist footwear: Primus Knit, Vivobarefoot. Laced upper. Wider sole and shore hardness OS 75 **Comparators:** Slip on casual shoe: Skechers go walk 4.0 pursuit (F) and superior 2.0—Jeveno (M). Barefoot	Standing with eyes open and closed Walking with single and dual task Timed up and go Star excursion balance	3D motion capture & plantar pressure analysis: Postural stability (movement of the centre of pressure during eyes open/closed); walking stability (margin of stability during normal/dual‐task walking); mobility (the timed up and go test and the star excursion balance test); perceptions of the shoes (monitor orthopaedic shoes questionnaire)
Cudejko, Gardiner, Akpan, and D’Août (2020b)	To investigate the effects of minimal shoes on postural and dynamic stability, and physical function in older people compared to conventional shoes. The secondary aims were: (i) To systematically explore the effects of a range of minimal footwear features on these factors and on perceptions, and (ii) to compare minimal shoes to barefoot.	United Kingdom	Cross‐sectional, repeated measures	Twenty‐two middle‐aged and older adults (mean age 55.4 years; SD 7.8 years); 11 female and 11 male	**Minimalist:** Minimalist shoe prototypes (11 iterations) of textured sole, toe spring, rubber hardness, width, split toe and ankle collar **Comparators:** Conventional shoes: Slip on casual shoe: Skechers go walk 4.0 pursuit (F) and superior 2.0—Jeveno (M). Barefoot, Minimalist CON: Conventional minimalist footwear: Primus Knit, Vivobarefoot. Laced upper. Wider sole and shore hardness OS 75	Standing Walking Timed up and go	3D motion analysis and plantar pressure analysis: (1) postural stability (movement of the centre of pressure), (2) walking stability, (3) mobility (the timed up and go test), and (4) perceptions of the shoes (monitor orthopaedic shoes questionnaire)
Jones et al. (2015)	Determine which conservative treatments (barefoot, shoes, and insoles) most lowers the EKAM during walking, to determine if any concurrent changes occur in the external knee flexion moment and to compare the degree of immediate knee pain reduction and comfort during usage	United Kingdom	RCT	Seventy adults aged 45+ with medial tibiofemoral osteoarthritis (mean age 60.3 years; SD 9.6 years); 27 female and 43 male.	**Minimalist:** Mobility shoe (defined in Shakoor, Lidtke, Sengupta et al. 2008): Flexible light‐weight shoe. Flexible polycarbon sole with grooves at major flexion points of foot **Comparators:** Shoe (laced casual oxford): Ecco. Flat sole. Two lateral wedges (5° LW) in ecco and mobility shoe (aim to mimic BF walking). Barefoot	Walking at a self‐selected speed	3D motion capture and force plate: External knee adductor moment (EKAM peaks and KAAI); external knee flexion moment; walking speed and knee pain Comfort questionnaire
Mazzella et al. (2024a)	Assess the immediate effect of a flat, flexible school shoe, when compared with a traditional school shoe, on knee joint kinematics and kinetics, and PFJ reaction forces during walking and running in adolescents with PFP.	Australia	Cross‐sectional, repeated measures	Twenty‐eight adolescents with PFP (mean age 14.3 years; SD 1.7 years); 12 female, 16 male.	**Minimalist: Flat flexible school shoe:** Vivobarefoot RA II. Zero‐heel toe offset and no stability or motion control features, mass = 180g. 23/25 minimalist index **Comparator**: Traditional school shoe: Clarks Daytona defined as stiff, rigid, 12 mm heel to toe offset, mass 350g. 2/25 minimalist index	Walking and running	3D motion capture & instrumented treadmill; knee kinetics and kinematics and PFJ reaction force Likert scale to measure comfort
Paterson et al. (2017)	Compare the effects of a range of flat flexible shoes to a range of more stable supportive shoes on parameters of the KAM in people with symptomatic medial knee OA. A secondary aim was to examine the effects of both shoe classes on the knee flexion Moment (KFM), as recent research suggests that this parameter may also be associated with medial tibiofemoral contact force	Australia	Cross‐sectional, repeated measures	Twenty‐eight adults with symptomatic knee osteoarthritis (mean age 63.7 years; SD 7.6 years); 13 female, 15 male.	**Minimalist:** Three minimalist footwear conditions (athletic, dress and casual); flat flexible heel thickness < 15 mm; shoe pitch < 10 mm; motion control absent; sole sagittal rigidity (rated using the ‘midfoot sole sagittal stability’ section of the footwear Assessment tool) minimal; shoe mass < 200 g based on US 9 **Comparator:** Three stable supportive footwear conditions (athletic, dress and casual). Heel thickness > 30 mm; shoe pitch > 10 mm; motion control present; sole sagittal rigidity rigid; shoe mass > 300 g based on US 9	Self‐selected walking	3D motion capture & force plate; knee EKAM, KAAI and flexor moment Knee pain
Ribeiro and João (2022)	To investigate the therapeutic effect of conservative treatment combining a custom insole with minimalist flexible shoes and the shoes alone in a gait‐training protocol, in the short and long term, in women with PF	Brazil	RCT	Thirty‐six women diagnosed with PF (mean age of minimalist group 46.4 ± 9.6 years, custom insole in shoe group 48.9 ± 9.8 years and control group 46.1 ± 10.7 years)	**Minimalist:** Minimalist flexible shoe, low‐cost double canvas, flat, walking shoe, without heel drop, a 5 mm anti‐slip rubber sole and a 3 mm internal wedge of ethylene vinyl acetate. Mass of between 91 and 182 g, depending on the shoe size. Minimalist shoe with custom orthotic **Comparator:** Control group (standard protocol with guidelines on footwear but not defined). Control group	Gait retraining programme	Heel pain (VAS pain scale); foot function (foot function index, foot health status questionnaire) and 6 min walk test Plantar pressure distribution (contact area, maximum force, peak pressure of ML RF, MF, FF, toes) Foot posture index
Ribeiro et al. (2022)	Investigate the effectiveness of mechanical treatment with customised insole and minimalist flexible footwear during gait training programme in women with calcaneal spur.	Brazil	RCT	Forty‐three women diagnosed with calcaneal spur; (mean age minimalist group 48.9 ± 9.4 years, custom insole in shoe group 50.3 ± 5.8 years, control group 47.8 ± 8.6 years	**Minimalist:** Low‐cost double canvas, flat, walking shoe: Moleca. 0 heel drop, 5 mm anti‐slip rubber sole and a 3 mm internal wedge of ethylene vinyl acetate. Mass between 91 and 182 g, depending on the shoe size. Minimalist footwear with customised orthopaedic insole **Comparator**: Control group (standard protocol with guidelines on footwear guidelines for walking but not defined)	Gait retraining programme	Heel pain (VAS pain scale); foot function (foot function index, foot health status questionnaire) and 6 min walk test. Plantar pressure distribution (contact area, maximum force, peak pressure of ML RF, MF, FF, toes)
Sacco et al. (2012)	To evaluate and compare the influence of a modern heeled shoe with the barefoot condition and inexpensive, flexible, nonheeled footwear (moleca), already produced and used on a large scale, on KAM during stair descent in elderly women with and without knee OA	Brazil	Case‐control	Thirty‐four women with OA (mean ± SD age 65 ± 6 years) and 17 controls without knee OA (mean ± SD age 66 ± 4 years)	**Minimalist**: Flat walking shoe without a heel, a thin 5‐mm anti‐slip flexible rubber sole and a 3‐mm internal wedge of ethylene vinyl acetate, with an upper body composed of double canvas. Mean (SD) weight is 0.172–0.019 kg, ranging from 0.142 to 0.193 kg depending on the size **Comparator:** Barefoot and own modern heeled shoe: An upper made of soft leather, a hard leather outsole (low flexibility) with a wide‐base heel	Stair descent—final step down from 5 step staircase	3D motion capture and force plates (in floor, not step): First KAM peak at the weight acceptance phase; KAM at the forward continuance phase; second KAM peak at the propulsion phase, and the non‐normalised adduction angular impulse
Shakoor, Lidtke, Wimmer et al. (2013)	To evaluate the effects of 6 months of use of this mobility footwear on gait kinematics and knee loading in participants with medial compartment knee OA	USA	Cohort	Sixteen adults with symptomatic knee OA (mean age 57 years; SD 10 years); 7 female and 9 male	**Minimalist:** A Mobility shoe defined as a flexible, lightweight shoe, with specialised grooves to allow for natural, barefoot‐like movement of the foot **Comparator:** Own footwear & barefoot condition. Own footwear varied by participant choice but were the shoes that the subjects had worn for walking tasks prior to entering the study	Walking overground, self‐selected and standardised (1 m/s) velocity	3D motion capture and force plates: External knee adductor moment and impulse; walking speed; stride length & frequency
Trombini‐Souza et al. (2020) ^a^	To elucidate which biomechanical factors are predictive of the reduction in first peak EKAM after 6‐month usage of the moleca shoe in older adult women with medial compartment knee OA	Brazil	RCT	Older women with knee OA (intervention group mean age 66 years, SD: 5 years; control group mean age 66 years, SD 6 years)	**Minimalist:** Flexible, flat and non‐heeled; low‐cost double canvas, flexible, flat, walking shoe without heels, a 5‐mm anti‐slip rubber sole and a 3‐mm flat insole of ethylene vinyl acetate **Comparator:** Own pair of a standard, neutral tennis shoe, without characteristics of a minimalist footwear and physically different to minimalist shoe	Overground walking, barefoot	3D motion capture and force plates: Frontal plane hip, knee and ankle angle, foot progression angle, medial‐lateral COP, frontal plane external hip, knee and ankle moments
Trombini‐Souza et al. (2015)^a^	To evaluate the therapeutic effect of a low‐cost, flexible, non‐heeled footwear on pain, self‐reported function, clinical (knee oedema and effusion) and gait‐biomechanical aspects of elderly women with medial knee OA	Brazil	RCT	Fifty older women with knee OA (intervention group mean age 66 years, SD: 5 years; control group mean age 66 years, SD 4 years)	**Minimalist:** Low‐cost double canvas, flexible, flat, walking shoe without heels, a 5‐mm anti‐slip rubber sole and a 3‐mm flat insole of ethylene vinyl acetate that provides only protection. The mean mass of the shoe was 0.172 kg (SD 0.019 kg; range 0.091–0.182 kg), depending on size **Comparator:** Own pair of a standard, neutral tennis shoe, without characteristics of a minimalist footwear and physically different to minimalist shoe	Overground walking, barefoot and in intervention (min) shoe	3D motion capture, force plates; WOMAC; algo‐functional lequesne index of severity of OA; 6‐min walk test
Xiang, Mei, Fernandez and Gu (2018)	Analyse the foot morphology (forefoot) deformation and plantar pressure changes through a 12‐week running intervention with the minimalist running shoes among males with mild and moderate hallux valgus	China	Cohort	Eleven adult males (mean age 26.2 years, SD3.1 years) with mild and moderate hallux valgus	Minimalist characteristics not reported No comparator	Barefoot walking (1.2 [0.2] m/s) and running (3.0 [0.2] m/s)	Easy‐foot‐scan—3D scanner; plantar pressure platform
Xiang, Mei, Wang et al. (2022)	To evaluate the longitudinal effect of minimalist footwear intervention on mild hallux valgus	China	Case study	1 male aged 26 years	Minimalist characteristics not reported No comparator	Walking, self‐selected velocity	Finite element modelling

^a^ Same study but different variables reported.

### 8.2 Footwear Characteristics

All but two studies reported footwear characteristics (see Table 1). Most of the studies (*n* = 11) compared minimalist footwear against a standard shoe, and three studies had an orthoses comparator, one study tested two versions of orthotic design both incorporating 5° lateral wedges (Jones et al. 2015), one study tested custom (flat) ethylene‐vinyl acetate (EVA) orthoses with 7 mm lateral wedges (Ribeiro and João 2022), and similarly, one used EVA devices with rear‐foot wedging (not defined) (Ribeiro et al. 2022). Characteristics of footwear varied across the studies and are reported in Table 1. They included running shoes/trainers (*n* = 5), casual shoes of various styles (*n* = 8) and barefoot (*n* = 3). Some studies included participants own footwear as a control condition (*n* = 3). The adaptation time offered for participants to familiarise themselves with new footwear also varied across the studies, although several studies did not report an adaptation time.

### 8.3 Outcome Measures

All studies featured instrumented data collection techniques, ranging from Inertial Measurement Units (IMU) to three‐dimensional gait analysis. Eleven studies featured three‐dimensional movement analysis, but the variables differed across the studies (see Table 2). With most studies focussing on the knee, it was unsurprising that knee kinetic and kinematic variables were frequently reported. Three studies adopted plantar pressure technology, and one study adopted finite element modelling. Walking at self‐selected pace was the most common functional task and reported in 12 studies, with variations such as single and dual tasking (Cudejko, Gardiner, Akpan, and D’Août 2020b), varied walking surfaces (Azhar et al. 2023), stair descent (Sacco et al. 2012) and gait retraining interventions (Ribeiro and João 2022). Four of the studies reported treadmill running (Bonacci et al. 2018, 2020; Mazzella et al. 2024; Xiang, Mei, Fernandez and Gu 2018).

**TABLE 2:** Study outcomes and mechanisms of action.

Study	Clinical application	Outcomes reported	Summary of findings	Mechanism of action
Azhar et al. (2023)	Balance and stability; falls	Balance (postural sway during bipedal and tandem standing) area over 30 s using gyko wearable sensor Walking stability area over 60 s using gyko wearable sensor Monitoring orthopaedic shoe questionnaire (MOS) (Van Netten et al. 2009). Perceptions of footwear on 100 mm VAS (attractiveness, comfort, fit, donning/doffing, heaviness) Comfort scale (footwear regions)	In minimalist v comparator: ↔ Balance (report non‐significant improvement in sway parameters) ↔ Walking stability (report non‐significant improvement in sway parameters) ↔ Comfort overall ↓ Heel‐cushioning, medio‐lateral control, arch height, heel cup fit, shoe heel & forefoot width	Footwear stability features may enhance balance and stability; enhanced somatosensory feedback may enhance stability
Bonacci et al. (2020)	Patellofemoral pain; variability	Joint angle variability hip knee and ankle. Flexion/Extension (flex/Ext) Adduction/Abduction (Add/Abd) Internal and external rotation (IR/ER) Joint moment variability hip, knee and ankle. Movement coupling variability: Thigh flex/Ext & tibial rotation Thigh Add/Abd & tibial rotation Thigh rotation & tibial rotation Tibial rotation & Ankle inv/Ev Thigh flex/Ext & tibial flex/Ext Thigh Add/Abd & Ankle inv/Ev Tibial rotation & Ankle Add/Abd	In minimalist v comparator: Joint angle variability: ↔ Hip flex/Ext ↔ Hip Add/Abd ↔ Hip IR/ER ↔ Knee flex/Ext ↔ Knee Add/Abd ↔ Knee IR/ER ↔ Ankle DF/PF ↔ Ankle inv/Ev ↔ Ankle IR/ER Joint moment variability: ↔ Hip flex/Ext ↔ Hip Add/Abd ↑ Hip IR/ER ↔ Knee flex/Ext ↑ Knee Add/Abd ↑ Knee IR/ER ↔ Ankle DF/PF ↔ Ankle inv/Ev ↔ Ankle IR/ER Movement coupling variability: ↔ Thigh flex/Ext & tibial rotation ↔ Thigh Add/Abd & tibial rotation ↔ Thigh rotation & tibial rotation ↔ Tibial rotation & Ankle inv/Ev ↔ thigh flex/Ext & tibial flex/Ext ↔ Thigh Add/Abd & Ankle inv/Ev ↔ Tibial rotation & Ankle Add/Abd	Increased variability may reduce repetitive joint loading and pain
Bonacci et al. (2018)	Patello‐femoral joint (PFJ) stress and joint reaction force (PFJRF)	Peak PFJ stress Peak PFJRF Peak knee extensor moment Peak knee flexion angle	All variables compared to running in the control shoe at preferred cadence, differences related to shoe only reported – not cadence. **Peak PFJ stress** ↓ In minimalist shoe at preferred cadence **Peak PFJRF** ↓ In minimalist shoe at preferred cadence **Peak knee extensor moment** ↓ In minimalist shoe at preferred cadence **Peak knee flexion angle** ↓ In minimalist shoe at preferred cadence	Minimalist footwear may influence knee kinematics and PFJ loading
Cudejko, Gardiner, Akpan, and D’Août (2020a)	Stability and mobility; falls	– Postural stability (movement of the CoP) – Mean velocity and the maximum range (ROM) of the CoP movement in anterior‐posterior (AP) and medial‐lateral (ML) direction). – Walking stability (margin of stability; MoS) – Timed‐up and go test (TUG) – Star excursion balance test (SEB) – MOS questionnaire perceptions of footwear on 100 m VAS (attractiveness, comfort, fit, donning/doffing, heaviness) Comfort scale (footwear regions)	In minimalist v comparator: ↓ CoP metrics (increased stability) ↓ AP ROM and velocity ↓ ML ROM and velocity ↑ MoS AP ↓ TUG completion time (better mobility) ↑ Reach distance for SEB in anterior, posterior, lateral and medial directions (better mobility) ↑ Fit and lightness	Conventional shoes alter posture and centre of mass. Minimalist shoes augmented stimulation of plantar mechanoreceptors
Cudejko, Gardiner, Akpan & D'Aout (2020^b^)	Stability and mobility; falls	– Postural stability (movement of the CoP) – Mean velocity and the maximum range (ROM) of the CoP movement in anterior‐posterior (AP) and medial‐lateral (ML) directions. – walking stability (movement of the CoP) mean velocity (mm/stance duration) and the maximum range (mm) of the COP displacement in the ML direction. TUG MOS questionnaire perceptions of footwear on 100 m VAS (attractiveness, comfort, fit, donning/doffing, heaviness) Comfort scale (footwear regions)	In minimalist v comparator: ↓ AP ROM and velocity ↓ ML ROM and velocity Therefore ↑ stability during standing (in all but two of the 11 minimalist shoes) ↓ ML ROM and velocity Therefore ↑ stability during walking (in four minimal conditions: Wider sole, high ankle collar, softer rubber and harder rubber). ↑ TUG in minimalist shoe with wider sole	Participants were more stable during standing when wearing each of the minimal shoes compared with conventional shoes. Minimalist shoes augmented stimulation of plantar mechanoreceptors
Jones et al. (2015)	Knee osteoarthritis	External knee adductor moment (EKAM peaks and KAAI). External knee flexion moment; walking speed Knee pain (5‐point likert scale) Comfort (10 cm VAS)	Control shoe used as reference group (except pain). In minimalist v comparator: **EKAM 1st peak** ↓ Barefoot walking ↓ For typical and supported wedge ↔ Mobility shoe **EKAM 2nd peak** ↔ Barefoot walking ↓ For typical and supported wedge ↔ Mobility shoe **KAAI** ↓ Barefoot walking ↓ For typical and supported wedge ↔ Mobility shoe **Knee flexor moment** ↓ Barefoot walking ↔ For other conditions **Comfort** ↓ Control shoe ↑For typical and supported wedge ↑Mobility shoe Knee pain ↓ supported wedge and mobility versus own shoe ↑ Control and barefoot versus own shoe **Walking speed** ↓ Barefoot walking ↑ With the mobility shoe	Reduce medial load to reduce pain in KOA
Mazzella et al. (2024a)	Reduce PFJ loading	Stride length Cadence Continuous waveform data across stride for (i) knee and ankle joint angles and moments in the sagittal plane, (ii) PFJ reaction force, and (iii) ankle joint power	**Minimalist school shoe v traditional school shoe** **Stride length** ↔ Walking ↓ Running **Cadence** ↔ Walking ↑ Running **Knee Angle** ↓ Flexion during walking (15%–35% of GC) ↓ Flexion during running (10%–33% of GC) **Ankle Angle** ↑ dorsiflexion during walking (0%–55% of GC) ↑ dorsiflexion during running (0%–30% of GC) **Knee moment** ↓ Extensor moment during walking (15%–40% of GC) ↓ Extensor moment during running (15%–25% of GC) **Ankle moment** ↑ plantarflexion moment during walking (5%–43% of GC) ↔ Plantarflexion moment during running **PJF reaction force** ↓ During walking (15%–40% of GC) ↓ During running (15%–25% of GC) **Ankle joint power** ↑ Peak ankle power during walking (40%–60% of GC). ↔ Peak ankle power during running	Reduce PFJ reaction force
Paterson et al. (2017)	Knee osteoarthritis	Peak EKAM in the first 50% of stance Positive KAAI Peak EKFM during stance phase moment Walking pain in knee (11‐point numerical rating scale)	**Peak EKAM** ↑ In all footwear conditions compared to barefoot but lower in flat flexible shoe styles **KAAI** ↑ In all footwear conditions compared to barefoot but lower in flat flexible shoe styles **Peak KFM** ↔ Between footwear conditions **Walking pain** ↔ Between footwear conditions	Reduce knee load
Ribeiro and João (2022)	Plantar fasciitis treatment	Primary outcomes VAS (heel pain) Foot function index (FFI) Foot health status questionnaire (FHSQ) 6‐min walk test Secondary outcomes Plantar pressure distribution (contact area, maximum force, peak pressure) at medial and lateral rear foot, midfoot, forefoot and toes.	**Compared to baseline assessment before intervention** **Primary outcomes** ↓ Calcaneus pain in minimalist shoe and minimalist with custom insole ↑ Foot function in both ↑ FHSQ in both ↑ 6‐min walk test in both **Secondary outcomes** Contact Area ↓ Forefoot in minimalist with custom insole ↓ Midfoot in both ↓ Medial and lateral rearfoot in minimalist with custom insole Maximum force ↓ Forefoot in minimalist with custom insole ↓ Midfoot in both ↓ Medial and lateral rearfoot in both Peak pressure ↓ Forefoot in both ↓ Midfoot with both ↓ Medial rearfoot with minimalist with custom insole ↓ Medial and lateral rearfoot with both	Intervention decreased the plantar pressure on the foot
Ribeiro et al. (2022)	Pain reduction in calcaneal spur.	Primary outcomes VAS (foot pain) Foot function (FFI, FHSQ‐Br, 6MWT) Comfort (not described) Secondary outcomes Plantar pressure distribution (contact area, maximum force, peak pressure) at medial and lateral rear foot, midfoot, forefoot and toes.	**Compared to baseline assessment before intervention** **Primary outcomes** ↓ Calcaneus pain in minimalist shoe and minimalist with custom insole ↑ Foot function in both ↑ FHSQ in both↑ 6‐min walk test in both ↓ Comfort in both **Secondary outcomes** Contact Area ↓ Forefoot in both ↓ Midfoot with minimalist ↓ Lateral rearfoot with minimalist Maximum force ↓ Forefoot with minimalist ↓ Midfoot in both Peak pressure ↓ Forefoot in both ↓ Midfoot with minimalist ↓ Medial and lateral rearfoot in both	Minimalist flexible footwear combined with customised orthopaedic insole could simulate barefoot gait and reduce vertical forces
Sacco et al. (2012)	Knee OA: reduce knee loading	First EKAM peak at weight acceptance (20% of stance phase) EKAM peak at forward continuance (horizontal body displacement of 20%–55% of stance phase) second E KAM peak at propulsion (80% of stance phase) Knee adduction (non‐normalised) angular impulse (KAAI).	Comparison between control group and OA group and heeled v barefoot in paper but not reported **First EKAM peak** ↓ For control and OA groups in minimalist v heeled shoe ↔ For control and OA groups in minimalist v barefoot **EKAM peak at forward continuance** ↓ For control and OA groups in minimalist v heeled shoe ↓ For control minimalist v barefoot ↔ For OA minimalist v barefoot **Second EKAM peak** ↓ For control and OA groups in minimalist v heeled shoe ↓ For control minimalist v barefoot ↔ For OA minimalist v barefoot **Knee adduction angular impulse (KAAI)** ↓ For control and OA groups in minimalist v heeled shoe ↓ For control minimalist v barefoot ↔ For OA minimalist v barefoot	The flexibility of barefoot locomotion reduces the external knee adduction moment (KAM), which is a biomechanical parameter that often increases in individuals with knee OA
Shakoor, Lidtke, Wimmer et al. (2013)	Knee OA; reduce knee joint moments and pain	Peak EKAM and KAAI. Walking speed. Stride length & frequency. Pain (WOMAC)	**Compared to baseline assessment before intervention** ↓ EKAM ↓ KAAI ↔ Knee flexion moments ↔ Walking speed ↓ Knee pain (WOMAC) *Additional time‐varying covariate analysis reported with shoes x intervention period*	Minimalist shoe shown to reduce knee loading. Also considers the potential for therapeutic gait adaptation with long‐term use of minimalist footwear
Trombini‐Souza et al. (2020)^a^	Reduced loading = reduced risk of OA development	Frontal plane hip, knee and ankle angle. Frontal plane external hip, knee and ankle moments. Foot progression angle, Medial‐lateral CoP offset from foot midline	**Intervention group compared to baseline assessment before intervention** ↓ Peak EKAM ↓ Knee frontal plane angle ↔ Hip front plane angle ↔ Ankle frontal plane angle ↔ Hip frontal plane moment ↔ Ankle frontal plane moment ↔ CoP offset ↔ Foot progression angle Outcomes for control group not reported	Three possible mechanisms underpinning the reduction in the first peak EKAM were presented: (1) distal neuromuscular factors; (2) reduction in the knee adduction angle; (3) no increase in hip adduction moment
Trombini‐Souza et al. (2015)^a^	Conservative treatment of knee OA.	Primary outcome Pain WOMAC score Second outcomes included Total WOMAC score Joint stiff and disability WOMAC scores Lequesne score 6‐min walk test EKAM (first peak) KAAI	After minimalist footwear intervention (comparison to baseline): ↓ WOMAC pain score ↑ WOMAC function score ↓ WOMAC stiffness score ↓ Overall WOMAC score ↓ Lequesne score ↔ 6‐min walk test ↔ EKAM first peak minimalist and barefoot ↓ KAM impulse in minimalist shoe ↔ KAAI in barefoot Control group data not reported.	Knee joint loads changed in both groups, but a longer intervention period was deemed necessary to modify these typical clinical signs of OA
Xiang, Mei, Fernandez and Gu (2018)	Hallux valgus; foot anatomical structure	Foot morphology Hallux abductus angle (HAA), hallux angle (HA), length, width, ball girth and waist girth Plantar pressure distribution: Peak pressure (PP), maximum force (MF), contact area (CA) and force time integral (FTI) distributed in big toe (BT), other toes (OT), metatarsal 1 (M1), metatarsal 2 (M2), metatarsal 3 (M3), metatarsal 4 (M4), metatarsal 5 (M5), medial midfoot (MM), lateral midfoot (LM) medial rearfoot (MR) and lateral rearfoot (LR). During walking and running	After minimalist footwear intervention (comparison to baseline) ↓ HAA ↓ HA ↔ Length ↓ Width ↔ Ball girth ↑ Waist girth ↓ PP and FTI at BT ↔ OT ↓ PP, MF and FTI at M1 ↑ PP at M2 ↑ PP and MF at M3 ↑ PP and MF at M4 ↔ M5 ↔ MM, ↔ LM ↔ MR ↑ CA at LR Walking data reported. See paper for running data.	Barefoot walking enabled the foot to achieve its biologically ‘normal’ morphology and functional performance
Xiang, Mei, Wang et al. (2022)	Hallux valgus; foot anatomical structure	Shape changes (Hausdorff distance and RMS) and metatarsophalangeal displacement von mises stress (MPa) distribution Minimal principal stress (MPa) distribution	Hausdorff distance and RMS approx. 0.3 and 0.28 mm) five metatarsals Varus realignment 1st MTP joint ↑ Displacement of the first MTP joint ↓ Stress loading across the metatarsals	Increased flexibility in the forefoot

*Note:* ↔, no significant change; ↑, significant improvement in minimalist shoe; ↓, significant reduction/deterioration in minimalist shoe.

Abbreviations: CIG, custom orthopaedic insoles combined with minimalist flexible shoes; COIG, customised orthopaedic insoles combined minimalist flexible footwear; EKAM, external knee adductor moment; KAAI, knee adductor angular impulse; PP, peak pressure; SG, minimalist flexible shoes; VAS, visual analogue scale; WOMAC, Western Ontario and McMaster Universities Osteoarthritis Index.

^a^ Same study but different variables reported.

Several functional measures featured across the studies and included the Timed‐Up and Go (Cudejko et al. 2020a, 2020b), Star Excursion Balance Test (Cudejko, Gardiner, Akpan, and D’Août 2020b), and six‐minute walk test (Ribeiro and João 2022; Ribeiro et al. 2022). Pain was a feature of the inclusion criteria for 9 studies (Bonacci et al. 2018, 2020; Jones et al. 2015; Mazzella et al. 2024; Paterson et al. 2017; Sacco et al. 2012; Shakoor, Lidtke, Wimmer et al. 2013; Trombini‐Souza et al. 2015, 2020) and measured as an outcome in seven studies. This was via a numerical rating scale in three studies (Jones et al. 2015; Ribeiro and João 2022; Ribeiro et al. 2022), and/or self‐report outcomes measures in six studies, the Foot Health Status Questionnaire (Bennett et al. 1998) was used in two studies (Ribeiro and João 2022; Ribeiro et al. 2022) and the Western Ontario and McMaster Universities Index (WOMAC) (Bellamy et al. 1988) in four studies (Paterson et al. 2017; Sacco et al. 2012; Shakoor, Lidtke, Wimmer et al. 2013; Trombini‐Souza et al. 2015). Participant report of footwear characteristics was presented in six studies and included footwear comfort (three studies) (Azhar et al. 2023; Jones et al. 2015; Mazzella et al. 2024) and aspects of the Monitor Orthopaedic Shoes questionnaire (Van Netten et al. 2009) such as attractiveness, fit, weight and ease of use (Azhar et al. 2023; Cudejko et al. 2020a, 2020b). Comfort was also reported by Ribeiro, de Souza & Joao (Ribeiro et al. 2022), but no measurement tool or procedure was defined.

The influence of minimalist footwear on outcomes varied across the studies and findings are summarised in Table 2. Based on the biomechanical outcomes, there was some evidence of minimalist footwear increasing variability of knee joint moments in PF pain (e.g. Bonacci et al. 2020); decreasing PFJ loading in PF pain (e.g. Bonacci et al. 2018); increasing stability, defined by a decrease in Centre of Pressure metrics coupled with an increase in Margin of Stability (e.g. Cudejko et al. 2020a, 2020b)) and decreasing knee moment magnitude (specifically EKAM and KAAI) compared with other shoes (e.g. Sacco et al. 2012; Sacco et al. 2012)). Evidence of impact on functional outcomes was demonstrated by increased stability (e.g. Cudejko et al. 2020a, 2020b)) although this was not consistent across studies with different variables reported (e.g. Azhar et al. 2023; Azhar et al. 2023)). Two of three studies reported an increase in the distance achieved in the 6‐min walk test (Ribeiro and João 2022; Ribeiro et al. 2022). For the clinical outcomes, studies demonstrated improved perception of footwear (e.g. fit and lightness) (Cudejko, Gardiner, Akpan, and D’Août 2020b), but findings about comfort varied when compared to other footwear (e.g. Azhar et al. 2023; Jones et al. 2015; Azhar et al. 2023; Jones et al. 2015)) and after a period of wear (Ribeiro et al. 2022). Similarly, changes in pain were not consistent across studies, with some reporting improvement (Trombini‐Souza et al. 2015) and others reporting no change (Paterson et al. 2017).

Reported mechanisms of action varied across studies and were not always tested. Mechanisms included somatosensory (Azhar et al. 2023; Cudejko et al. 2020a, 2020b), reduced movement variability (Bonacci et al. 2020), reduction in joint stress or loading through kinematic or kinetic parameters (Bonacci et al. 2018; Jones et al. 2015; Mazzella et al. 2024; Paterson et al. 2017; Shakoor, Lidtke, Wimmer et al. 2013; Trombini‐Souza et al. 2015), distal neuromuscular factors (Trombini‐Souza et al. 2020), and reduced plantar loading (Ribeiro and João 2022; Ribeiro et al. 2022).

## 9 Discussion

The role of footwear as a clinical intervention or adjunct to intervention is contentious and the purpose of this scoping review was to explore the potential for minimalist footwear in the treatment and/or rehabilitation of lower limb impairments across the life course. Our review has mapped the clinical populations where minimalist footwear has been tested and we have identified 16 studies, with most focussing on knee pathology, specifically knee osteoarthritis. Our review has also identified the biomechanical, functional, and clinical variables reported in studies. Biomechanical variables were most reported but there was variation across studies, along with differences in testing protocol(s) and footwear choices. Collectively, there was evidence demonstrating the impact of minimalist footwear on biomechanical, functional and clinical outcomes, but there were differences with clinical populations, outcome measures, and settings for data collection.

Knee joint pathology (e.g. osteoarthritis) was the most common clinical problem and biomechanical outcomes were typically reported, indicating the potential for minimalist footwear to influence kinematic and kinetic parameters at the knee. However, the study samples were relatively small, and interventions varied in their design, dose and dose response. Given the breadth of the outcomes reported in the literature, the clinical recommendations that can be drawn are limited and a systematic review of studies is warranted. Footwear is an important self‐management strategy for adults with knee problems and the need for clearer footwear recommendations for people with knee osteoarthritis (Moseng et al. 2024) remains. We acknowledge that it is unlikely that a *one size fits all* approach to footwear choice is appropriate, but mechanistic studies are important to establish if minimalist footwear is beneficial and influences clinical outcomes (Campbell et al. 2019). Additionally, exploring the characteristics of responders and non‐responders to minimalist footwear may help to establish more personalised footwear recommendation models.

Aside from the literature on therapeutic or prescribed footwear for foot problems seen in conditions such as Rheumatoid Arthritis (Frecklington et al. 2018; Tehan et al. 2019; Tenten‐Diepenmaat et al. 2018) and Diabetes (Jarl et al. 2020; Jorgetto et al. 2019; Luo et al. 2022), studies have reported on the benefits of footwear foot pain and disability in people with Gout (Frecklington et al. 2019), and foot pain (Menz et al. 2015). In this review, there was limited evidence exploring minimalist footwear for foot conditions, but our search identified several trial registrations for studies currently recruiting or have completed recruitment. As outlined in the results, we have identified 4 studies reporting the benefits of minimalist footwear for foot conditions, 2 on heel pain and two on hallux valgus. Similar to the work on knee pathology, footwear is an important self‐management strategy, but with such limited evidence, it is unclear if minimalist footwear is an effective intervention (or adjunct) for people with foot pain. We acknowledge that studies have reported the benefits of minimalist footwear for improving foot strength, but unless reporting on interventions for clinical populations, these studies have not featured in our scoping review (Curtis et al. 2021; Jaffri et al. 2023). The benefits of targeting intrinsic muscle strength have been reported (Wei et al. 2022), but some caution about the clinical benefits across different populations is needed. In practice, footwear is a notoriously challenging concept to navigate (Carter et al. 2016; Matthias et al. 2021; Tehan et al. 2019) and successful influence on footwear behaviour is challenging for clinicians (Jarl et al. 2020). Footwear parameters such as aesthetics and comfort were included in some studies and are important as these represent important dimensions influencing footwear decisions. Both parameters are complex concepts (Menz and Bonanno 2021) but offer little insight into the potential longer‐term adherence to an intervention.

Sensorimotor and mechanical effects were reported in studies exploring the impact of footwear on balance and gait characteristics, but neurophysiological parameters appear to be a notable omission from these studies. The focus on foot health outcomes and plantar pressure parameters is important but the plantar skin is a complex neurophysiological organ (Riabova et al. 2024) and advances in understanding the influence of footwear on neurophysiological parameters are needed to underpin credible arguments about footwear choice(s) as an intervention, and to support translation into practice. It is 10+ years since the publication of a review on footwear interventions on balance, performance and gait in older adults (Hatton et al. 2013), where the need for longitudinal studies focussing on neurophysiological output was proposed. This gap in the literature remains an important barrier and we advocate for interdisciplinary collaborations to advance footwear science and clinical interventions.

### 9.1 Study Limitations

We have presented a scoping review and the findings from our review are intended to map the breath of studies that have been conducted on minimalist footwear as an adjunct to intervention. We acknowledge that our design limits the appraisal of existing evidence, and we have made no comment on the quality of the evidence. The exclusion of studies conducted in asymptomatic participants is a key limitation of this search and has potentially omitted some key studies. Equally, we imposed language restrictions on our search and have included only studies published in English, which has introduced a publication bias.

### 9.2 Conclusion

Through exploring the clinical potential of minimalist footwear, we have sought to advance discussion about footwear interventions for foot and lower limb impairments across the life course. Our review has outlined the biomechanical, clinical and functional variables reported in studies and concludes that there are limited studies focussing on the clinical benefits of minimalist footwear interventions. Across the evidence included in this search, there are inconsistencies across clinical populations and methodological approaches. The characteristics reported in this review may prove to be beneficial for informing future research.

## Author Contributions

**Stewart C. Morrison** **:** conceptualization, methodology, investigation, formal analysis, supervision, project administration, data curation, writing – original draft, writing – review and editing, funding acquisition. **Carina Price** **:** conceptualization, methodology, investigation, formal analysis, project administration, data curation, writing – original draft, writing – review and editing. **Ben Langley** **:** conceptualization, methodology, investigation, formal analysis, project administration, data curation, writing – original draft, writing – review and editing. **Binyu Luo** **:** formal analysis, data curation, methodology, writing – review and editing.

## Ethics Statement

The authors have nothing to report.

## Conflicts of Interest

The authors declare no conflicts of interest.

## Supporting information

Supporting Information S1

## Data Availability

Data sharing not applicable to this article as no datasets were generated or analysed during the current study.
