# Supplementary material for: Minimalist Footwear in the Treatment and Rehabilitation of Lower Limb Impairments Across the Life Course: A Scoping Review
Source: Musculoskeletal Care. 2025 May 24;23(2):e70122. doi: 10.1002/msc.70122 (PMC12103103; doi:10.1002/msc.70122)
Supplement: Supplementary file 1 — Supporting Information S1 [file MSC-23-e70122-s001.docx]

**Appendix I: Search strategy**

Search strategy for MEDLINE, conducted July 2024.

1 "Minimalist Footwear".mp. 62

2 ("Minimalist Shoe" or "Minimal shoe" or Shoe*OR shod or barefoot).mp. [mp=title, book title, abstract, original title, name of substance word, subject heading word, floating sub-heading word, keyword heading word, organism supplementary concept word, protocol supplementary concept word, rare disease supplementary concept word, unique identifier, synonyms, population supplementary concept word, anatomy supplementary concept word] 2254

3 1 or 2 2280

4 exp Therapeutics/ 5353306

5 (Treatment* or Therap* or Prevention* or Injur* or Intervention* or Restor* or Rehabilitation).mp. [mp=title, book title, abstract, original title, name of substance word, subject heading word, floating sub-heading word, keyword heading word, organism supplementary concept word, protocol supplementary concept word, rare disease supplementary concept word, unique identifier, synonyms, population supplementary concept word, anatomy supplementary concept word] 13145916

6 4 or 5 14702586

7 ("Functional Impairment" or Disabilit* or Pain or Fall* or balance or Trip* or stability or Limitation* or Dysfunction).mp. [mp=title, book title, abstract, original title, name of substance word, subject heading word, floating sub-heading word, keyword heading word, organism supplementary concept word, protocol supplementary concept word, rare disease supplementary concept word, unique identifier, synonyms, population supplementary concept word, anatomy supplementary concept word] 3850524

8 3 and 6 and 7 407

9 limit 8 to (english language and humans and yr="2000 -Current") 303

10 qualitative research.ab. 18454

11 reviews.ti. 12626

12 conference.ti. 34873

13 10 or 11 or 12 65871

14 9 not 13 303

Search strategy for CINAHL, conducted July 2024.

S1 minimal* N2 footwear

S2 minimal* N2 shoe

S3 barefoot

S4 shoe* or shod

S5 S1 OR S2 OR S3 OR S4

S6 Treatment* or Therap* or Prevention* or Injur* or Intervention* or Restor* or Rehabilitation

S7 (MH "Persons with Disabilities")

S8 (MH "International Classification of Functioning, Disability, and Health") OR "functional impairment"

S9 (MH "Pain") OR (MH "Stability") OR (MH "Balance, Postural")

S10 "physical limitations"

S11 S7 OR S8 OR S9 OR S10

S12 S5 AND S6 AND S11

S13 TI qualitative research or qualitative study

S14 TI reviews

S15 TI conference

S16 S13 OR S14 OR S15

S17 S12 not S16

S18 S12 not S16 Limiters - Publication Date: 20000101-20241231

S19 S12 not S16 Limiters - Publication Date: 20000101-20241231; English Language
